# Integrative analysis of lung molecular signatures reveals key drivers of idiopathic pulmonary fibrosis

**DOI:** 10.1186/s12890-021-01749-3

**Published:** 2021-12-07

**Authors:** Sung Kyoung Kim, Seung Min Jung, Kyung-Su Park, Ki-Jo Kim

**Affiliations:** 1 Division of Pulmonology, Department of Internal Medicine, St. Vincent’s Hospital, College of Medicine, The Catholic University of Korea, Seoul, Republic of Korea; 2 Division of Rheumatology, Department of Internal Medicine, St. Vincent’s Hospital, College of Medicine, The Catholic University of Korea, Seoul, Republic of Korea; 3 Division of Rheumatology, Department of Internal Medicine, St. Vincent’s Hospital, The Catholic University of Korea, 93 Jungbu-daero, Paldal-gu, Suwon, Gyeonggi-do 16247 Republic of Korea

**Keywords:** Idiopathic pulmonary fibrosis, Unsupervised clustering, Key driver genes

## Abstract

**Background:**

Idiopathic pulmonary fibrosis (IPF) is a devastating disease with a high clinical burden. The molecular signatures of IPF were analyzed to distinguish molecular subgroups and identify key driver genes and therapeutic targets.

**Methods:**

Thirteen datasets of lung tissue transcriptomics including 585 IPF patients and 362 normal controls were obtained from the databases and subjected to filtration of differentially expressed genes (DEGs). A functional enrichment analysis, agglomerative hierarchical clustering, network-based key driver analysis, and diffusion scoring were performed, and the association of enriched pathways and clinical parameters was evaluated.

**Results:**

A total of 2,967 upregulated DEGs was filtered during the comparison of gene expression profiles of lung tissues between IPF patients and healthy controls. The core molecular network of IPF featured p53 signaling pathway and cellular senescence. IPF patients were classified into two molecular subgroups (C1, C2) via unsupervised clustering. C1 was more enriched in the p53 signaling pathway and ciliated cells and presented a worse prognostic score, while C2 was more enriched for cellular senescence, profibrosing pathways, and alveolar epithelial cells. The p53 signaling pathway was closely correlated with a decline in forced vital capacity and carbon monoxide diffusion capacity and with the activation of cellular senescence. CDK1/2, CKDNA1A, CSNK1A1, HDAC1/2, FN1, VCAM1, and ITGA4 were the key regulators as evidence by high diffusion scores in the disease module. Currently available and investigational drugs showed differential diffusion scores in terms of their target molecules.

**Conclusions:**

An integrative molecular analysis of IPF lungs identified two molecular subgroups with distinct pathobiological characteristics and clinical prognostic scores. Inhibition against CDKs or HDACs showed great promise for controlling lung fibrosis. This approach provided molecular insights to support the prediction of clinical outcomes and the selection of therapeutic targets in IPF patients.

**Supplementary Information:**

The online version contains supplementary material available at 10.1186/s12890-021-01749-3.

## Background

Idiopathic pulmonary fibrosis (IPF) is a chronic interstitial lung disease characterized by progressive scarring of the lung parenchyma associated with a steady worsening of respiratory symptoms and a decline in pulmonary function, ultimately leading to death [1]. IPF is currently treated with systemic antifibrotic drugs, such as pirfenidone and nintedanib, which have been shown to delay the progressive decline of lung function and to reduce mortality [2]. However, neither pirfenidone nor nintedanib block or reverses the progression of IPF. Although therapeutic interventions targeting the immune response, inflammation, or oxidative stress have been attempted, none have been proven to be successful, and no effective pharmacotherapy for IPF exists yet [3–5]. These unsatisfactory results in drug development might be partially ascribed to the complexity and heterogeneity of IPF. The active cellular components and mechanistic features of inflammation and fibrosis are mixed, and their levels differ depending on the disease status or anatomical lesions [1, 6, 7]. Further, IPF is associated with diverse clinical progressions, from an asymptomatic stable state to gradual progressive respiratory failure or rapid deterioration of respiratory function through acute exacerbation [8].

Molecular subgroups exist within some fibrotic diseases in which different clinical phenotypes or outcomes are presented [9–12]. Gene expression profiling has provided insights into the pathogenesis of IPF [6, 8, 13, 14]. In previous pilot studies, molecular signatures from lung parenchyma proved helpful in predicting the likelihood of disease progression and therapeutic responsiveness [13, 15]. However, because all previous studies have been performed with cohorts of variable sizes and with different patient backgrounds, technical protocols, and technologies, direct comparisons between datasets and results are not feasible. A comprehensive integrated analysis of a compendium compiled using genome-wide datasets could reduce dataset bias, capture features missing from previous studies, and detect key factors driving the disease.

In this study, we compiled lung tissue transcriptome datasets from public data repositories to establish an IPF compendium and characterized the cellular and molecular features in detail. The samples were separated using data-driven, unsupervised clustering methods, and the clustered subgroups were subjected to prognostic profiling. Finally, we employed an integrative network-based approach and Bayesian inference to identify key drivers of the disease and evaluated the impact of current and investigational drugs in the context of the disease module.

## Methods

### Systematic search and data collection

We used the keywords “idiopathic pulmonary fibrosis,” “interstitial lung disease,” “lung,” “transcriptomics, microarray, or RNA-sequencing,” “dataset” in Google Scholar and PubMed to find relevant publications on the topic of lung gene signatures of patients with IPF. We retrieved all publications that were accompanied by high-throughput datasets and ultimately selected 13 datasets with the GEO series (GSE) IDs GSE10667, GSE21369, GSE24206, GSE32537, GSE35145, GSE47460, GSE53845, GSE72073, GSE83717, GSE99621, GSE110147, GSE124685 and GSE150910 (Additional file 1: Table S1). The combined datasets included 585 IPF patient samples and 362 normal healthy controls and covered 15,447 genes in common.

### Data normalization and removal of batch effects

For one-channel arrays, the Robust Multi-array Average (RMA) method (R package affy) was applied to the image data of a set of replicates for background correction, normalization, and probe-set summarization [16]. For dual-channel arrays, the image data were imported, background correction was performed using normexp (R package limma), and red and green channels were separated [17, 18]. The matrix vectors were normalized using quantile normalization [19]. Residual technical batch effects arising due to heterogeneous data integration were corrected using the ComBat function [19]. Quality assurance and distribution bias was evaluated using principal component analysis. After preprocessing, systematic and dataset-specific bias was greatly reduced (Additional file 2: Figure S1).

### Filtering of differentially expressed genes

In order to identify the differentially expressed genes (DEGs), we employed three independent methods: (a) an empirical Bayesian method (eBayes) using the Benjamini–Hochberg procedure with adjusted *p* value < 0.01 as the significance threshold (R package limma) [20]; (b) the Significance Analysis of Microarray (SAM) method, with false discovery rate (FDR) < 0.01 as the significance threshold (R package EMA) [21]; (c) multivariate inferential analysis method, with false discovery rate (FDR) < 0.01 as the significance threshold (R package acde) [22]. An absolute value of fold change > 1.5 was considered as DEGs. The resulting list of DEGs is the intersection of the three individual DEGs sets for each method to minimize the FDR statistic (Additional file: Figure S2). Upregulated DEGs were used in all the subsequent analyses.

### Pathway- and cell subset-driven enrichment analysis

We performed a functional enrichment analysis focusing on upregulated DEGs using Enrichr software [23]; adjusted *p* value was made using the Benjamini–Hochberg method for correction for multiple hypotheses testing and the terms were considered significant if the adjusted *p* value was less than 0.05. For biological processes or signaling pathways, gene-set enrichment analysis (GSEA) was conducted using Broad Institute software to assess overrepresentation [24]. Gene-set information on signaling pathways or biological processes was obtained from the Kyoto Encyclopedia of Genes and Genomes (KEGG), Gene Ontology, and the Reactome databases [25–27]; the terms were regarded as significant if the false discovery rate was lower than 0.25, per the interpretative guidelines [24]. The enrichment score (ES) reflects the degree to which a gene set is overrepresented at the top or bottom of a ranked list of genes. GSEA calculates the ES by walking down the ranked list of genes, increasing a running-sum statistic when a gene is in the gene set and decreasing it when it is not. The magnitude of the increment depends on the correlation of the gene with the phenotype. The ES is the maximum deviation from zero encountered in walking the list. Since ES can be biased by a single permutation, ES is adjusted based on the gene set enrichment scores for all dataset permutations, producing normalized enrichment score (NES). NES is used to compare analysis results across gene set by accounting for differences in gene set size and in correlations between gene sets and the expression dataset [24]. The enrichment results were visualized with the Enrichment Map format, where nodes represent gene sets and weighted links between the nodes represent an overlap score depending on the number of genes two gene sets share (Jaccard similarity coefficient) [28]. To intuitively identify redundancies between gene sets, the nodes were connected if their contents overlap by more than 10%.

To test for gene enrichment in individual samples, we used a single-sample gene-set enrichment analysis, which defines an enrichment score as the degree of absolute enrichment of a gene set in each sample within a given dataset [29]. Information on markers of cell populations in the human lung was taken from a recent single-cell RNA sequencing study [30]. The digital signature algorithm was applied to deconvolve the expression of a tissue into the component profiles of each cell type using a set of marker genes that are highly expressed in each cell type [31]. The eigengene score for a specific gene-set group was calculated as previously described [32].

### Construction of protein–protein interaction network

To assess the interconnectivity of DEGs in the IPF lung samples, we constructed a protein–protein interaction network based on the human interactome database [33]. Graph theory concepts such as degree, closeness, and betweenness were employed to assess the topology of this network [34, 35]. The degree centrality is defined as the number of node neighbours. The betweenness centrality measures the node’s role in acting as a bridge between separate clusters by computing the ratio of all shortest paths in a network that contains a given node. The closeness centrality quantifies how fast a given node in a connected graph can access all other nodes; hence, the more central a node is, the closer it is to all other nodes. Hub molecules were defined as shared genes within the top 10% with the highest rank in each arm of the three centrality parameters [36].

### Unsupervised clustering and determination of the optimal number of clusters

To categorize the IPF patients into subgroups based on their molecular signatures, agglomerative hierarchical clustering was performed with the dissimilarity matrix given by Euclidean distance and Ward’s method was used to join similar clusters [37]. To interpret the robustness of each clustering output for multiple models of one to six clusters (*k*)*,* we computed the silhouette scores and the within-cluster sum of squared error for each *k* [38]. The maximum peak of the silhouette score plots and the point at which the sum of squared error begins to diminish (the “elbow” method) determined the optimal number of clusters. *t*-distributed stochastic neighborhood embedding was used to confirm the unsupervised clustering results [39], a powerful dimensionality reduction method that captures data variance by attempting to preserve the distances between data points from high to low dimensions without any prior assumptions regarding data distribution.

### Key driver analysis

When mapped onto the protein–protein interaction network, disease-associated genes tend to co-localize and form networks of functionally related genes called disease modules. To predict genes that regulate the disease module, we performed a key driver analysis (KDA) using a previously defined algorithm that mathematically identifies causal modulators of the regulatory state of functionally interconnected gene groups (R package mergeomics) [40]. The significance of key driver genes (KDGs) for a given gene set in a particular Bayesian network was estimated by permuting the gene labels in the network and estimating the *p* value based on the simulated null distribution. False discovery rates were estimated using the Benjamini–Hochberg method and genes with values below 0.01 were considered key drivers.

### Network-based diffusion scoring

To quantify the leverage of each gene in the disease module, we employed label propagation and the network diffusion algorithm, which models heat flow from the seed genes through interactions in a protein–protein interaction network. The *z*-scaled Monte-Carlo method loaded in the R package diffuStats was used to calculate the diffusion score of the nodes based on the genes across the largest connected component of the network with default parameters [41].

### Statistical analysis

For continuously distributed data, between-group comparisons were performed using the unpaired *t*-test. Categorical or dichotomous variables were compared using Fisher’s exact test. Correlation analysis between variables was carried out using Pearson’s method and Bonferroni correction. All analyses were conducted in R (version 4.0.3, The R Project for Statistical Computing, www.r-project.org).

## Results

### Differentially expressed genes and their network and enriched pathways

A total of 2,967 upregulated DEGs was filtered during the comparison of gene expression profiles of lung tissues between IPF patients and healthy controls (Additional file 2: Figure S2). PPI networks constructed using those DEGs identified 6,658 interactions and 1,190 genes with more than one linkage to other genes. The network included *DSP* gene, a variant of which are known to confer the risk of developing IPF [42, 43], and 14 biomarkers (*CCL18*, *CD28*, *CHI3L1*, *CLU*, *CXCL13*, *HSPA4*, *KRT19*, *MMP1*, *MMP7*, *MUC16*, *POSTN*, *SPP1*, *TNFSF13B*, and *VCAM1*) [2, 44, 45]. The largest connected component (LCC), also known as the giant component, is the connected component of a network that contains a significant proportion of all network nodes [46]. The LCC is typically the most complex part of the network and represents the network’s core. Here, the LCC consisted of 1,777 genes. Centrality analysis revealed 172 hub molecules, which included one susceptibility genes (*DSP*) and three biomarkers (*HSPA4*, *SPP1*, and *VCAM1*).

**Fig. 1:**
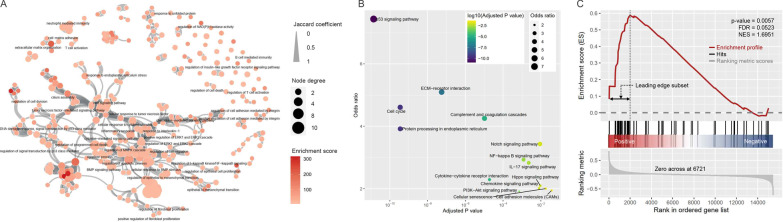
**A** Enrichment map from the functional enrichment analysis using the Enrichr tool. Nodes represent gene ontology–biological process (GO-BP) gene sets, and GO-BPs of interest are labeled. Their color intensity and size are proportional to the enrichment score and the degree, respectively. The edge thickness represents the degree of overlap between gene sets, and only edges with a Jaccard similarity coefficient larger than 0.10 are visualized. **B** Functional enrichment analysis of DEGs for KEGG pathways. Point size and color express the odds ratios and adjusted *p* values. **C** GSEA plot of the p53 signaling pathway

We performed a functional enrichment analysis of the LCC-DEGs, obtained 327 Gene Ontology biological process terms (Fig. 1A), and identified key enriched KEGG pathways (Fig. 1B): the p53 (*P* = 1.37 × 10^−11^) and PI3K-Akt (*P* = 7.15 × 10^−4^). The most significant pathway, p53 signaling pathway, was confirmed by GSEA (*p* value = 0.0057, FDR = 0.0523, and NES = 1.6951) (Fig. 1C).

### Gene expression-driven subgrouping and pathobiological characterization

**Fig. 2:**
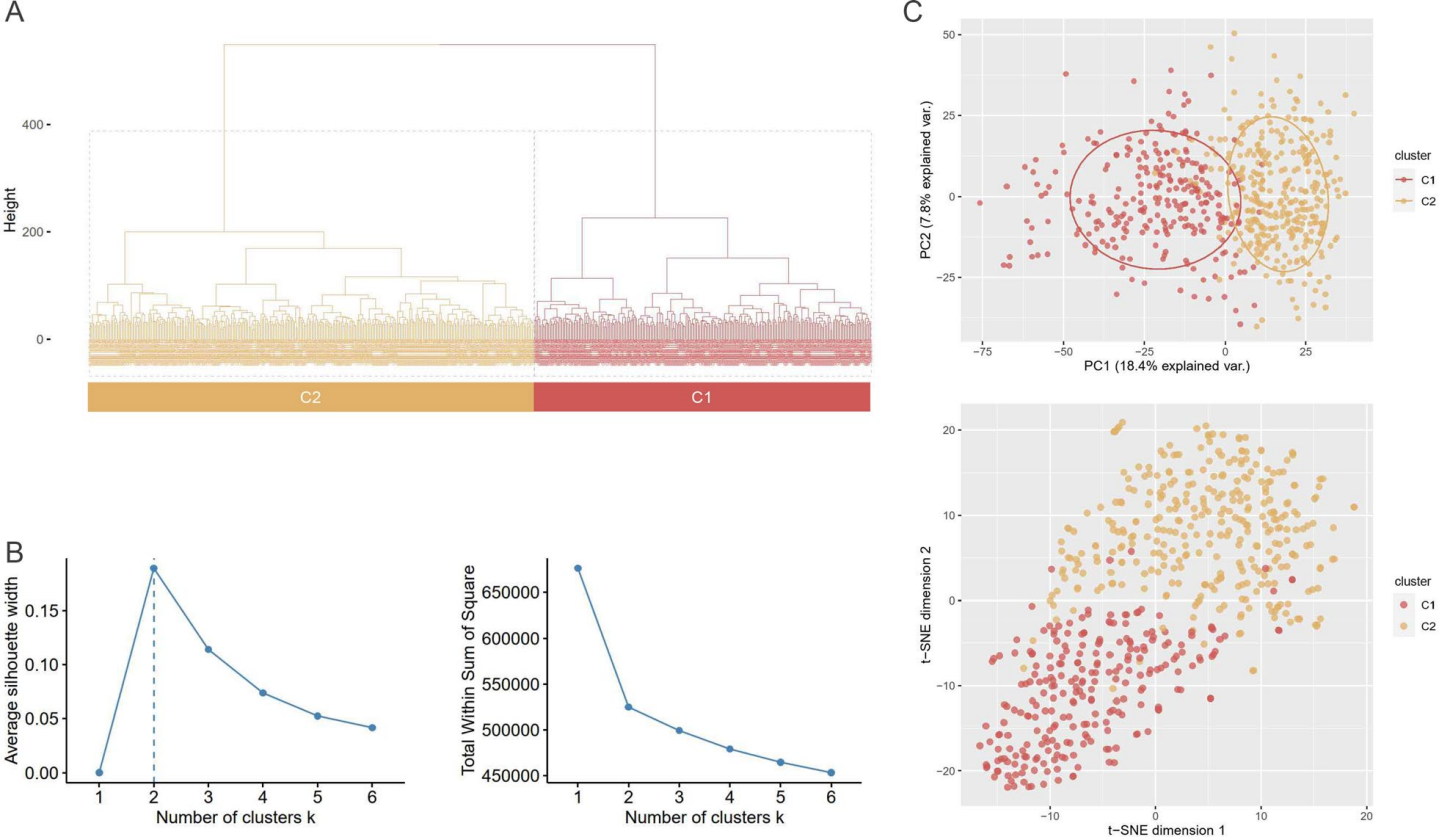
Unsupervised clustering using an agglomerative hierarchical clustering method. **A** Dendrogram of the hierarchical clustering based on differentially expressed genes (DEGs). **B** Silhouette scores and the sum of squared error were used to identify the optimal number of clusters. The maximum peak of the silhouette score plots and the point at which the sum of squared error begins to diminish (the “elbow” method) determined the optimal number of clusters. **C** *t*-distributed stochastic neighbor embedding (*t*-SNE) and principal component analysis of the DEG profiles. C1 (n = 252) and C2 (n = 333) are colored red and yellow, respectively

To identify gene expression-driven subgroups in an unbiased manner, we performed an agglomerative hierarchical clustering using DEG profiles from lung tissue samples of 585 IPF patients. We found that two clusters most optimally represented the data by computing the silhouette score and sum of squared error for two to six clusters (Fig. 2A). Two clustered subgroups were designated C1 (n = 252) and C2 (n = 333) in order. Segregation of IPF subgroups were reproduced by *t*-stochastic neighbor embedding analysis and principal component analysis (Fig. 2C).

**Fig. 3:**
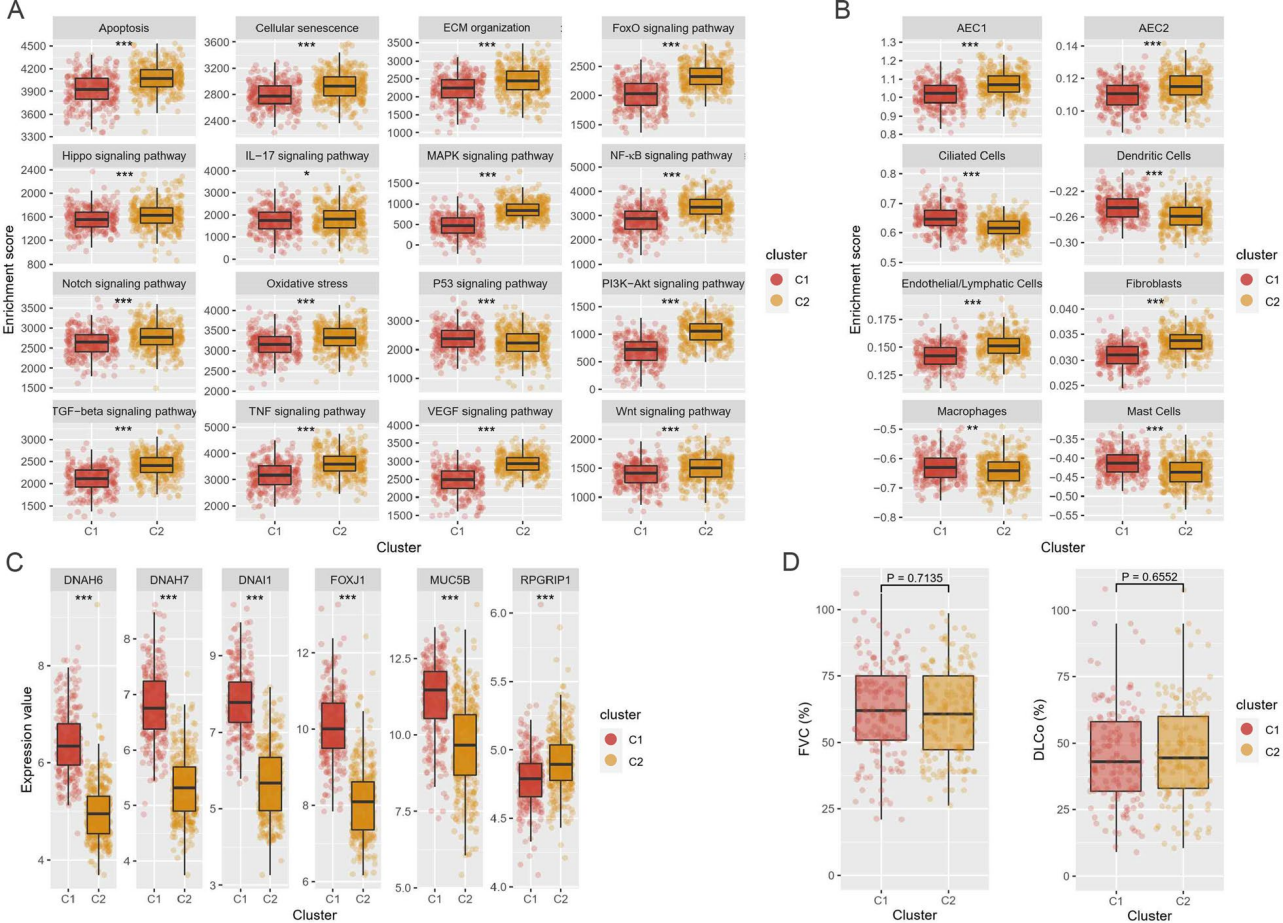
Pathway and cell subset-driven characterization. **A** Pathway enrichment scores of IPF subgroups. **B** Cell subset enrichment score of IPF subgroups. Cell scores predict relative enrichment for cell types. **C** Expression levels of cilia-associated genes and ciliated cell markers. **D** Forced vital capacity (FVC) and diffusing capacity of the lung for carbon monoxide (DL_CO_). Pulmonary function testing was performed within 6 months before lung tissue sampling, and the data on FVC and DL_CO_ was available for 328 patients (C1 = 160, C2 = 168). Differences across the two subgroups were evaluated using an unpaired *t*-test. *P < 0.01; **P < 0.01; ***P < 0.001

To distinguish pathobiological characteristics among the two subgroups, we curated IPF-related pathways and imported information on markers of cell populations in the human lung from the literature [1, 7, 30] and compared the enrichment scores of pathways and cell subsets. The p53 signaling pathway was more enriched in C1 (*P* = 2.803 × 10^−4^), while cellular senescence, FoxO signaling pathway, PI3K-Akt signaling pathway, and TGFβ signaling pathway were more activated in C2 (Fig. 3A and Additional file 2: Figure S3). Ciliated and dendritic cells were more enriched in C1 (*P* = 3.43 × 10^−21^ and *P* = 5.07 × 10^−14^, respectively), whereas alveolar epithelial cells type I (AEC1), alveolar epithelial cells type II (AEC2), fibroblasts and macrophages were more populated in C2 (Fig. 3B). Cilium-associated genes (*DNAH6*, *DNAH7*, and *DNAI1*) and ciliated cell markers (*FOXJ1* and *MUC5B*) were also highly expressed in C1 (Fig. 3C) [13, 47]. These results indicated that C1 was more transformed to microscopic honeycombing state [13, 48]. However, there was no difference in forced vital capacity (FVC) and carbon-monoxide diffusion capacity (DL_CO_) between the two groups (Fig. 3D).

### Association of enriched pathways with pulmonary functional parameters

**Fig. 4:**
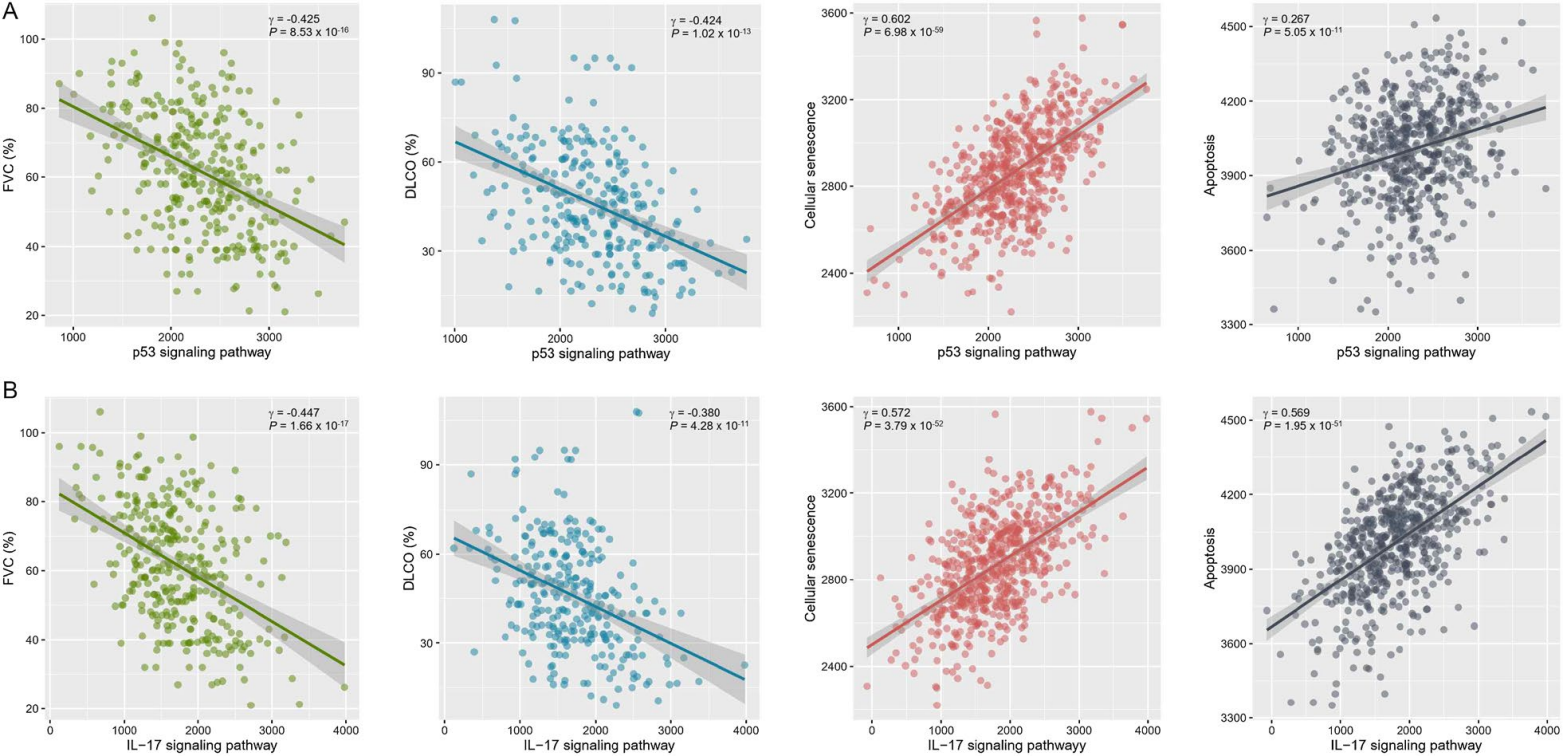
**A** Correlation of p53 signaling pathway with forced vital capacity (FVC), diffusing capacity of the lung for carbon monoxide (DL_CO_), cellular senescence and apoptosis. **B** Correlation of IL-17 signaling pathway with forced vital capacity (FVC), diffusing capacity of the lung for carbon monoxide (DL_CO_), cellular senescence and apoptosis. Correlation between variables was investigated using Pearson’s method and Bonferroni correction

A decline in the FVC and DL_CO_ of lungs is associated with a more severe IPF prognosis [49]. We examined correlations between enriched pathways and pulmonary function test parameters (Additional file 2: Figure S4). FVC (% predicted) and DL_CO_ (% predicted) was most strongly correlated with the p53 and IL-17 signaling pathway (n = 328, all γ < -0.35, *P* < 1.0 × 10^–10^) (Fig. 4 and Additional file 2: Figure S4). The p53 and IL-17 signaling pathway was also positively correlated with cellular senescence (n = 328, all γ > 0.54, *P* < 2.2 × 10^–16^) and apoptosis (n = 328, all γ > 0.24, *P* < 4.2 × 10^–5^) (Fig. 4).

### Prognostic risk estimation using molecular biomarkers

**Fig. 5:**
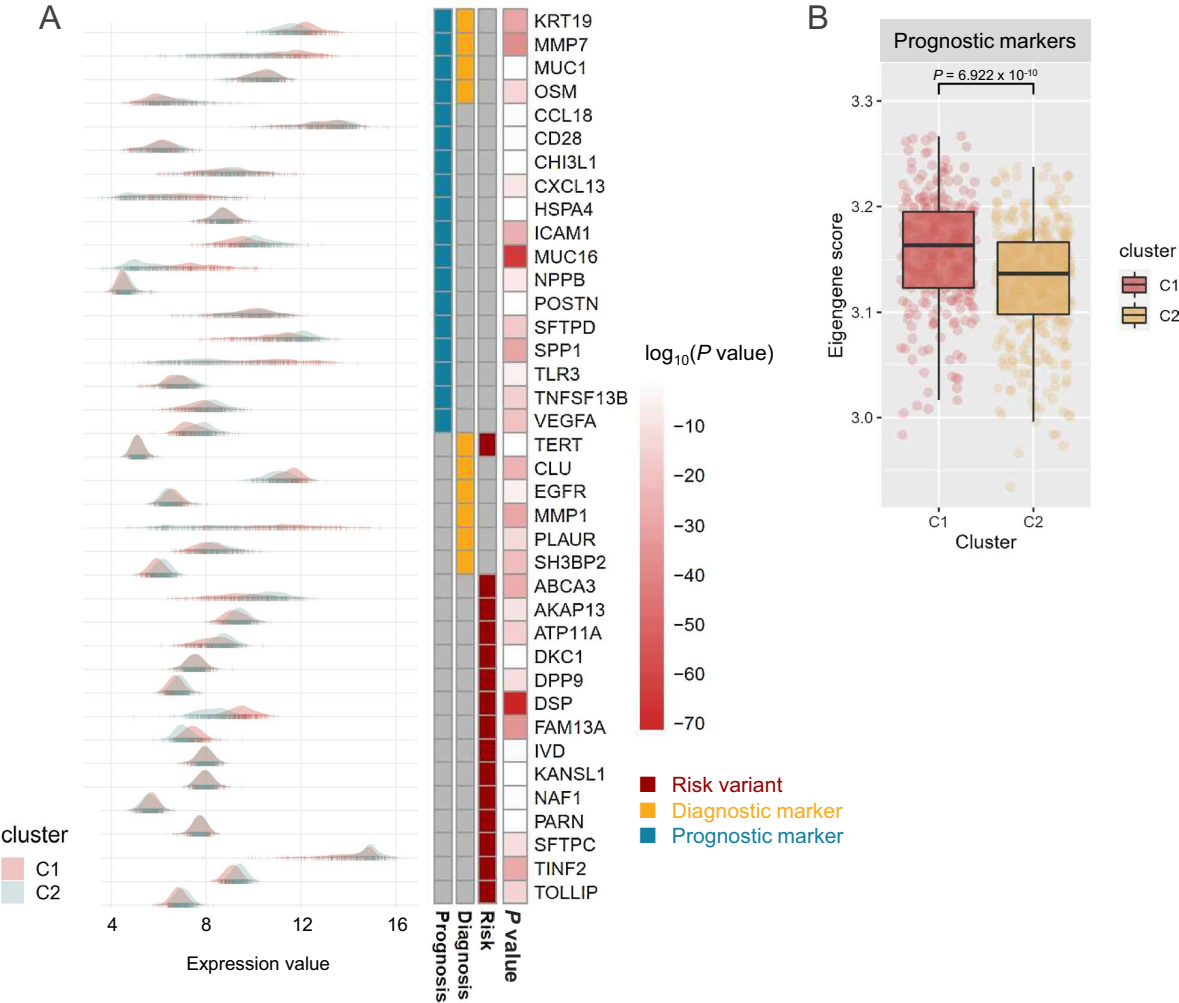
IPF biomarker expression within the two subgroups. **A** Expression values of genetic risk variants and diagnostic and prognostic markers. **B** Eigengene scores of prognostic markers within IPF subgroups. The difference between the subgroups was evaluated using an unpaired *t*-test

IPF biomarkers could allow improved clinical classification and guide diagnostic, therapeutic, and prognostic approaches to enable disease management [2, 44, 45]. In total, 38 molecular biomarkers were identified in the gene expression profiles of IPF lung tissue and were categorized into three subgroups: risk variants (n = 15), diagnostic markers (n = 10), and prognostic markers (n = 18). Gene expression values in 30 biomarkers differed significantly between the two subgroups (Fig. 5A), and 11 genes (*CLU*, *CXCL13*, *DSP*, *EGFR*, *FAM13A*, *KRT19*, *MMP1*, *MMP7*, *MUC16*, *NAF1*, *SPP1*) were more highly expressed in C1 than in C2. The most differentially expressed gene in C1 was *DSP* (*P* = 4.15 × 10^−72^, fold change 2.20), which was followed by *MUC16* (*P* = 2.64 × 10^−64^, fold change 3.65), a strong predictor for disease progression and mortality in IPF [50]. This was confirmed by a comparative DEG analysis of the subgroups (Additional file 2: Figure S5). The integrated levels of biomarkers were determined using eigengene scores based on categorized biomarker panels [2, 32, 44]. The prognostic eigengene scores of subgroup C1 were significantly higher than those in C2 (*P* = 6.922 × 10^−10^) (Fig. 5B) as confirmed by gene-set enrichment analysis (Additional file 2: Figure S6).

### Key drivers of the disease module

**Fig. 6:**
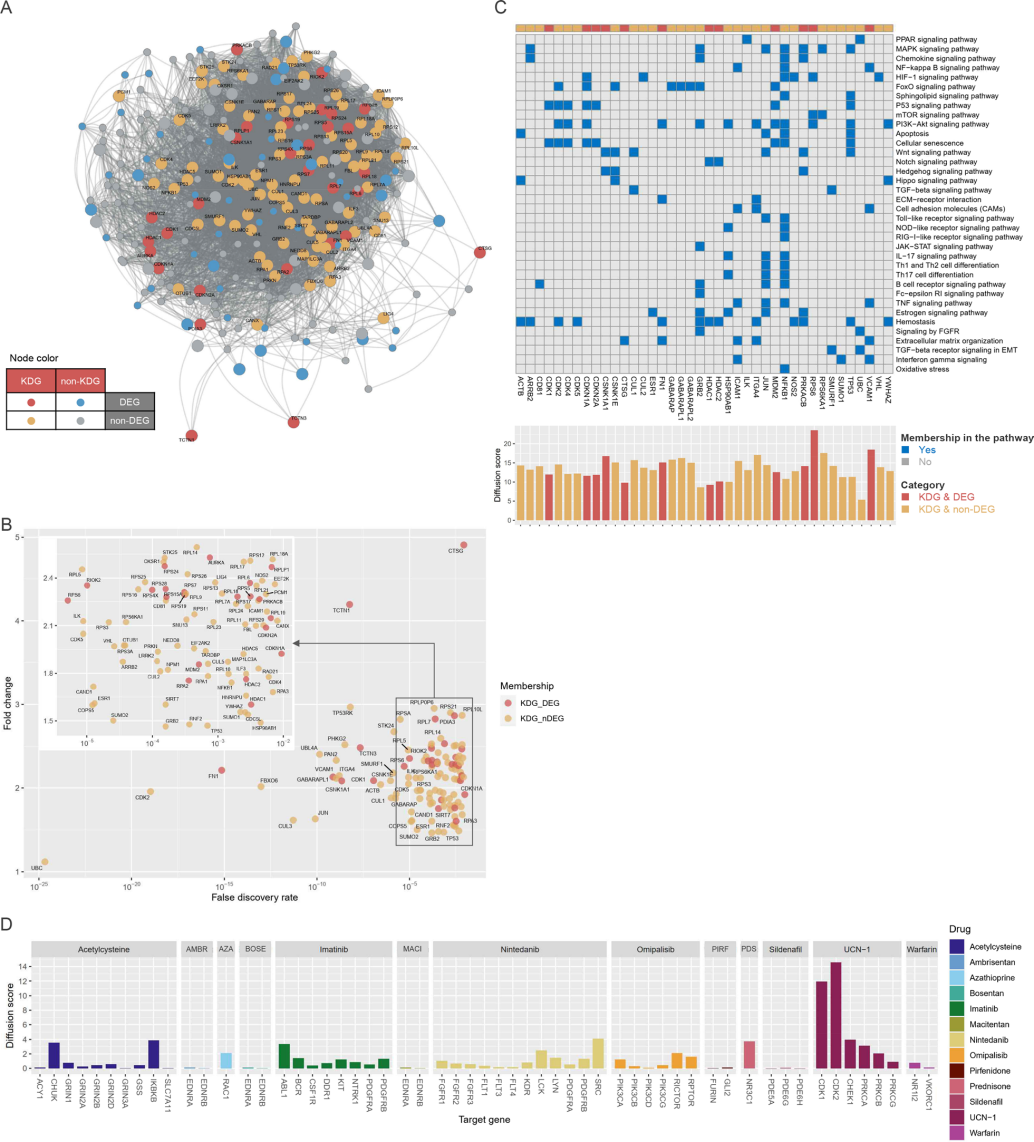
Key driver analysis and kernel-based diffusion scores. **A** Probabilistic causal gene network projection and key driver analysis to identify causal regulators in the IPF disease module. Key driver genes (KDGs) and their neighbors are distinguished by color. **B** Fold change and false discovery rate of KDGs. **C** KDGs involving IPF-associated pathways (upper panel) and their diffusion scores (lower panel). **D** Current and investigational drugs for IPF and their molecular targets and diffusion scores. Diffusion scores were calculated using the *z*-scaled Monte-Carlo method based on the largest connected component of the disease module

Elucidation of linkages within a disease module can lead to the identification of KDGs that are predicted to modulate the regulatory state of the module and are particularly suitable for prioritizing as causative of disease development and progression [40]. We constructed a Bayesian network by projecting the LCC-DEGs onto the human interactome and employed KDA. We identified 119 KDGs, of which 30 were DEGs (Fig. 6A, B). The diffusion kernel ranks a gene using the sum of a global distance measure and diffusion rate from all seed genes of a disease [41]. To better understand genes’ leverage within the core disease module, we calculated the network-based diffusion score for the KDGs belonging to IPF-associated pathways using the network kernel diffusion algorithm (Fig. 6C) [41]. RPS6 (diffusion score = 23.545) was top-ranked by diffusion score and was followed by RPS6KA1(21.509), VCAM1(18.475), ITGA4(17.047), and CSNK1A1(16.772). FN1(15.088), ICAM1(15.472), CDK2(14.546), CDK1(11.926), CDKN2A(11.846), and CKDN1A(11.586) also ranked as high-priority genes.

We collected information on the target molecules of 11 current treatment drugs and two investigational drugs (omipalisib, UCN-01) from a publicly available database and literature [2, 51–53] and compared their diffusion scores (Fig. 6D). The target molecules of *N*-acetylcysteine, azathioprine, imatinib, nintedanib, and prednisone were scored higher than those of endothelin receptor blockers, pirfenidone, sildenafil, and warfarin. Notably, UCN-01, an ATP competitive inhibitor, targeted the cyclin-dependent kinases (CKDs) and checkpoint kinase 1 (CHEK1) with high diffusion scores. CDK1 and CDK2 were KDGs and key elements of the p53 signaling pathway and cellular senescence.

## Discussion

In the present study, we built a comprehensive transcriptomic compendium of IPF lung tissue and performed an integrative analysis to better understand the relationship between cellular and molecular expression patterns and clinical parameters. An unsupervised cluster analysis of the IPF transcriptomic profiles yielded two subgroups with different cellular and pathologic activities and prognostic risk profiles. Finally, we identified KDGs and molecules that may serve as promising targets for therapeutic intervention based on network-based Bayesian inference.

Biological functions in living organisms are orchestrated by the cooperative interactions of genes, proteins, and chemical compounds. Likewise, a complex disease is rarely the consequence of an abnormality in a single gene but rather results from the aberrant activation of pathways or disease modules by dysregulated genes and their linked neighbors. We identified the p53 signaling pathway as the most significant dysregulated pathway in IPF, and its enrichment score had a close correlation with cellular senescence and apoptosis in the lung tissue of IPF patients. This finding aligns with previously reported upregulation of p53 and activation of the p53 signaling pathway in response to the proliferation and hyperactivity of AECs leading to AEC apoptosis and senescence, the pathologic hallmarks of IPF lungs [1, 54, 55]. IL-17 was also localized to active area of IPF and profibrotic roles of IL-17 in the pre-clinical models were well-documented [56]. Given that the p53 and IL-17 signaling pathways were closely correlated with FVC and DL_CO_, p53 and IL-17 signaling pathway activity could be an intriguing biomarker bridging the mechanistic feature and clinical condition in IPF.

We identified two novel molecular subtypes of IPF using an unsupervised clustering method. C1 subgroup was more enriched with p53 signaling pathway and ciliated cell signature, indicating further transformation into fibrosed structural change, honeycombing state. Histologically, honeycomb cysts are lined with ciliated cells that express a variety of epithelial markers [57] and this result are consistent with the previous results [13, 48]. Interestingly, C1 highly expressed *MMP7*, *MUC16*, and *SPP1*, which were powerful predictors of IPF progression [15, 50]. This underscores a close relationship between the p53 signaling pathway, AEC senescence, and progressive disease in IPF, suggesting the need for a stratified approach to patient management based on the molecular signature of lung tissue. Cellular senescence, oxidative stress, and profibrosing signaling pathways including FoxO-, PI3K-Akt-, TGFβ-, and Wnt signaling pathways were more enriched in C2 subgroup, and molecular signatures of AECs were also stronger, indicating that C2 subgroup is less advanced and under ongoing fibrosis. If the fibrotic processes are effectively controlled, it is presumed that C2 subgroup has partial reversibility.

Current IPF therapies cannot effectively modify the disease’s clinical course and their efficacy is inconsistent, although the anti-fibrotic drugs pirfenidone and nintedanib have demonstrated to ability to significantly slow respiratory deterioration in some IPF patients [1, 2]. Their limited and heterogeneous efficacy might be partly ascribed to a failure to optimally target pathways that will disrupt the IPF disease module. Therefore, we constructed differentially expressed and probabilistic causal gene networks to model molecular interactions and causal gene relationships and applied a Bayesian network-based analysis to identify key drivers of the IPF disease module. *CDK1*, *CDK2*, *CDKN1A*, *CDKN2A*, and *MDM2* were identified as KDGs involving both the p53 signaling pathway and cellular senescence. To better understand the KDGs in the disease module network, we calculated their diffusion scores. Fibronectin (FN1) is responsible for mediating cell–matrix adhesion and is essential in driving myofibroblast differentiation. Inhibition of FN1 deposition attenuated fibrosis in hepatic and cardiac fibrosis models [58, 59]. FN1 was highly ranked in our analysis but seemed to be of low druggability because it is an end product of multiple fibrosing pathways deposited at an extracellular matrix. In contrast, CDKs and HDACs are fascinating targets because they are high-priority drivers, and drugs targeting these genes are currently in use or under clinical trials as anti-cancer agents. The unbalanced proliferation and profibrosing activity of AEC2 and fibroblasts is a key initial event in the pathogenesis of IPF [1]. CDKN1A, also known as p21, is a physiologic CDK antagonist under the control of p53 and was also identified as a KDG. In the bleomycin-induced pulmonary fibrosis model, the forced expression of p21 exerted both anti-apoptotic and anti-fibrotic effects [55, 60]. HDAC inhibitors are known to cause cell-cycle arrest by inducing CDKN1A or inhibiting CDKs and effectively suppress profibrotic fibroblast phenotypes IPF, notably offering better performance than that of pirfenidone [61]. These results could be more applicable to the high-risk C1 subgroup than to subgroup C2.

To evaluate the use of current drug therapies in the disease module, we compared the diffusion scores of their target molecules. *N*-acetylcysteine, imatinib, and nintedanib ranked higher than did other drugs and were assumed to be more effective owing to their multiple targets. The performance of pirfenidone was likely underestimated because its exact targets and mechanisms are not clearly defined. Notably, the investigational drug UCN-01 (7-hydroxystaurosporine) showed good diffusion scores for its targets. UCN-01 targets CDK1, CDK2, and CHEK1, the main components of the p53 signaling pathway and cellular senescence, and reactivates FoxO3 to control its inappropriate proliferation and differentiation [52, 53]. In particular, UCN-01 showed great promise in the pre-clinical IPF model by reverting the IPF myofibroblast phenotype in vitro and blocking the bleomycin-induced lung fibrosis in vivo [53].

This study had several limitations. First, the combination of multiple datasets inevitably caused the loss of genes that overlapped only among some datasets, and the correction of the batch effect was not ideal. Second, we did not address the association with clinical factors, such as radiographic pattern or fibrosis score, due to the lack of this information. Third, minority signatures of specific cell subsets might have been diluted because the gene expression signature was at the bulk tissue level. Fourth, the datasets did not provide detailed background medications for individual patients. Surgical lung biopsy protocol [62, 63], the fresh lung area that best represents the disease should be biopsied for reliable results. Lung tissue under ongoing fibrosis reflects the current pathologic status rather than the response to treatment. Although some molecular signatures could be susceptible to the effect of current or past treatments and potentially biased, it is considered that they would not be enough to overturn our finding of the overwhelming significance of the p53 signaling pathway.

## Conclusion

IPF represents a major medical challenge with high unmet treatment needs. Our network-based integrative approach described discrete IPF subtypes with distinct cellular and molecular characteristics and revealed their significance in terms of clinical prognostic scales. Patient stratification could be leveraged to formulate customized therapies and improve clinical trial design. KDGs and target molecules were identified in the defined disease module, and particularly, inhibition against CDKs or HDACs afforded great promise of successful anti-fibrotic drugs. This not only explained the limitations of current pharmacotherapies but also provided insights into navigating new drug therapies.

## Supplementary Information

**Additional file 1: Table S1.** A summary of the datasets used in the study.

**Additional file 2: Figure S1.** Principal component analysis on the compendium of IPF lung tissue transcriptomics before and after normalization and batch correction. **Figure S2.** Refinement of the DEGs. **Figure S3.** Pathway enrichment scores according to IPF subgroups. **Figure S4.** Correlation between pulmonary function parameters, pathway, and cell subset enrichment score. **Figure S5.** Volcano plot of expressed genes between two subgroups. **Figure S6.** Enrichment scores of prognostic markers according to IPF subgroups by gene-set enrichment analysis.

## Data Availability

All datasets used in this study have been previously published and are accessible via the Gene Expression Omnibus (GEO) website (https://www.ncbi.nlm.nih.gov/geo/, accessed on 10 January 2021) using the GSE accession numbers listed in the Additional file 1: Table S1. All data generated or analyzed during this study are included in this published article [and its additional information files].

## Abbreviations

AEC1: Alveolar epithelial cells type I
AEC2: Alveolar epithelial cells type II
DEG: Differentially expressed gene
DLCO: Carbon-monoxide diffusion capacity
eBayes: Empirical Bayesian method
FDR: False discovery rate
FVC: Forced vital capacity
GSE: GEO series
IPF: Idiopathic pulmonary fibrosis
KDA: Key driver analysis
KDG: Key driver genes
KEGG: Kyoto Encyclopedia of Genes and Genomes
LCC: Largest connected component
RMA: Robust Multi-array Average
SAM: Significance Analysis of Microarray
