# Supplementary material for: Integrative analysis of lung molecular signatures reveals key drivers of idiopathic pulmonary fibrosis
Source: BMC Pulm Med. 2021 Dec 7;21:404. doi: 10.1186/s12890-021-01749-3 (PMC8650281; doi:10.1186/s12890-021-01749-3)
Supplement: Supplementary file 1 — Additional file 1: Table S1. A summary of the datasets used in the study. [file 12890_2021_1749_MOESM1_ESM.pdf]

Integrative analysis of lung molecular signatures reveals key drivers of idiopathic pulmonary fibrosis

Sung Kyoung Kim[1*], Seung Min Jung[2*], Kyung-Su Park[2], Ki-Jo Kim[2]

[1] Division of Pulmonology, Department of Internal Medicine, St. Vincent's Hospital, College of Medicine, The Catholic University of Korea, Seoul, Republic of Korea

[2] Division of Rheumatology, Department of Internal Medicine, St. Vincent's Hospital, College of Medicine, The Catholic University of Korea, Seoul, Republic of Korea

* Both authors contributed equally to this work.

Supplementary Table

Table S1.

(A) A summary of the datasets used in the study

Dataset	Reference	Country	Platform	IPF	NC	Lung tissue sampling	Source
GSE10667	*Am J Respir Crit Care Med*. 2009;180:167-175.[1]	USA	Affymetrix	31	15	Explanted lung biopsy or warm autopsy	
GSE21369	*BMC Med Genomics*. 2011;4:8.[2]	USA	Illumina	11	6	Surgical lung biopsy or lungs explanted during lung transplantation	LTRC
GSE24206	*BMC Med Genomics*. 2011;4:70.[3]	USA	Agilent	17	6	Surgical lung biopsy or lungs explanted during lung transplantation	
GSE32537	*Thorax*. 2013;68:1114-1121.[4]	USA	Agilent	119	50	Surgical lung biopsy or lungs explanted during lung transplantation	LTRC
GSE35145	*Am J Respir Crit Care Med*. 2012;186:525-535.[5]	USA	Agilent	4	4	Surgical lung biopsy or lungs explanted during lung transplantation	LTRC
GSE47460	*Am J Respir Crit Care Med*. 2016;194:1392-1402.[6]	USA	Affymetrix	160	108	Surgical lung biopsy or lungs explanted during lung transplantation	LTRC
GSE53845	*Thorax*. 2015;70:48-56.[7]	USA	Illumina	40	8	Surgical lung biopsy or lungs explanted during lung transplantation	
GSE72073	*Respir Res*. 2015;16:124.[8]	China	Illumina	5	3	Surgical lung biopsy	
GSE83717	*BMC Pulm Med*. 2017;17:15.[9]	USA	Affymetrix	6	5	Surgical lung biopsy or lungs explanted during lung transplantation	LTRC
GSE99621	*Clin Immunol*. 2018;325:1-13.[10]	USA	Illumina	18	8	Lungs explanted during lung transplantation	
GSE110147	*Respir Res*. 2018;19:153.[11]	Canada	Affymetrix	22	11	Lungs explanted during lung transplantation	
GSE124685	*JCI Insignt*. 2019;4:e131597.[12]	USA	IonTorrent	49	35	Lungs explanted during lung transplantation	LTRC
GSE150910	*Am J Respir Crit Care Med*. 2020;202:1430-1444.[13]	USA	Illumina	103	103	Surgical lung biopsy or lungs explanted during lung transplantation	LTRC
Total				585	362		

IPF, idiopathic pulmonary fibrosis; LTRC, Lung Tissue Research Consortium; NC, normal controls.

(B) A demographic summary of the patients with IPF used in the study

Dataset	Number of IPF samples	Sex Male (%)	Age (mean)	Smoking (%)	Race	FVC% (mean)	DLCO% (mean)
GSE10667	31	22 (70.9)	68.1	20 (64.5)	Caucasian 31	68.6	48.3
GSE21369	11	8 (72.7)	58.1	NA	Caucasian 10<br>African-American 1	62.7	NA
GSE24206	17	12 (70.6)	64.2	NA	NA	60.7	44.3
GSE32537	119	77 (64.7)	62.6	70 (58.8)	Caucasian 111<br>Other 8	61.2	45.1
GSE35145	4	3 (75.0)	60.3	NA	Caucasian 4	NA	NA
GSE47460	160	110 (68.8)	64.1	99 (61.9)	Caucasian 146<br>African-American 8<br>Asian 3<br>Hispanic 2<br>Other 1	64.3	48.0
GSE53845	40	32 (80)	70	29 (72.5)	NA	70.3	49.6
GSE72073	5	5 (100)	51.1	NA	NA	NA	NA
GSE83717	6	NA	NA	NA	NA	NA	NA
GSE99621	18	18	71.1	4	Caucasian 18	44.8	35.5
GSE110147	22	17 (77.3)	62	NA	NA	57	37
GSE124685	49	49	57	49	NA	58.7	27.6
GSE150910	103	57 (55.3)	60.3	55 (53.4)	Caucasian 85<br>Hispanic 7<br>Asian 2<br>African-American 4<br>Other 4	56.2	40.8

DLCO, Diffusing capacity of the Lung for carbon monoxide; FVC, forced vital capacity; IPF, idiopathic pulmonary fibrosis; NA, not available

(C) A demographic summary of the normal subjects used in the study

	GSE32537	GSE47460	GSE150910	Others
Number	50	108	103	101
Age, mean (year)	47.5	63.6	59.9	
Sex, male (%)	27 (54)	49 (45)	45 (44)	
Race, Caucasian (%)	41 (82)	100 (92)	NA	
Smoking (%)	28 (56)	65 (59.9)	53 (55)	
FVC%, mean	NA	94.4	72.0	
DLCO%, mean	NA	84.1	97.7	

References

1 Konishi K, Gibson KF, Lindell KO*, et al.* Gene expression profiles of acute exacerbations of idiopathic pulmonary fibrosis. *Am J Respir Crit Care Med* 2009;180:167-75.

2 Cho JH, Gelinas R, Wang K*, et al.* Systems biology of interstitial lung diseases: integration of mRNA and microRNA expression changes. *BMC Med Genomics* 2011;4:8.

3 Meltzer EB, Barry WT, D'Amico TA*, et al.* Bayesian probit regression model for the diagnosis of pulmonary fibrosis: proof-of-principle. *BMC Med Genomics* 2011;4:70.

4 Yang IV, Coldren CD, Leach SM*, et al.* Expression of cilium-associated genes defines novel molecular subtypes of idiopathic pulmonary fibrosis. *Thorax* 2013;68:1114-21.

5 Sanders YY, Ambalavanan N, Halloran B*, et al.* Altered DNA methylation profile in idiopathic pulmonary fibrosis. *Am J Respir Crit Care Med* 2012;186:525-35.

6 Tan J, Tedrow JR, Dutta JA*, et al.* Expression of RXFP1 Is Decreased in Idiopathic Pulmonary Fibrosis. Implications for Relaxin-based Therapies. *Am J Respir Crit Care Med* 2016;194:1392-402.

7 DePianto DJ, Chandriani S, Abbas AR*, et al.* Heterogeneous gene expression signatures correspond to distinct lung pathologies and biomarkers of disease severity in idiopathic pulmonary fibrosis. *Thorax* 2015;70:48-56.

8 Geng J, Huang X, Li Y*, et al.* Down-regulation of USP13 mediates phenotype transformation of fibroblasts in idiopathic pulmonary fibrosis. *Respir Res* 2015;16:124.

9 Vukmirovic M, Herazo-Maya JD, Blackmon J*, et al.* Identification and validation of differentially expressed transcripts by RNA-sequencing of formalin-fixed, paraffin-embedded (FFPE) lung tissue from patients with Idiopathic Pulmonary Fibrosis. *BMC Pulm Med* 2017;17:15.

10 Luzina IG, Salcedo MV, Rojas-Peña ML*, et al.* Transcriptomic evidence of immune activation in macroscopically normal-appearing and scarred lung tissues in idiopathic pulmonary fibrosis. *Cell Immunol* 2018;325:1-13.

11 Cecchini MJ, Hosein K, Howlett CJ*, et al.* Comprehensive gene expression profiling identifies distinct and overlapping transcriptional profiles in non-specific interstitial pneumonia and idiopathic pulmonary fibrosis. *Respir Res* 2018;19:153.

12 McDonough JE, Ahangari F, Li Q*, et al.* Transcriptional regulatory model of fibrosis

progression in the human lung. *JCI Insight* 2019;4.

13 Furusawa H, Cardwell JH, Okamoto T*, et al.* Chronic Hypersensitivity Pneumonitis, an Interstitial Lung Disease with Distinct Molecular Signatures. *Am J Respir Crit Care Med* 2020;202:1430-44.
