# Supplementary material for: Integrative analysis of lung molecular signatures reveals key drivers of idiopathic pulmonary fibrosis
Source: BMC Pulm Med. 2021 Dec 7;21:404. doi: 10.1186/s12890-021-01749-3 (PMC8650281; doi:10.1186/s12890-021-01749-3)
Supplement: Supplementary file 2 — Additional file 2: Figure S1. Principal component analysis on the compendium of IPF lung tissue transcriptomics before and after normalization and batch correction. Figure S2. Refinement of the DEGs. Figure S3. Pathway enrichment scores according to IPF subgroups. Figure S4. Correlation between pulmonary function parameters, pathway, and cell subset enrichment score. Figure S5. Volcano plot of expressed genes between two subgroups. Figure S6. Enrichment scores of prognostic markers according to IPF subgroups by gene-set enrichment analysis. [file 12890_2021_1749_MOESM2_ESM.pdf]

Integrative analysis of lung molecular signatures reveals key drivers of idiopathic pulmonary fibrosis

Sung Kyoung Kim[1*], Seung Min Jung[2*], Kyung-Su Park[2], Ki-Jo Kim[2]

[1] Division of Pulmonology, Department of Internal Medicine, St. Vincent's Hospital, College of Medicine, The Catholic University of Korea, Seoul, Republic of Korea

[2] Division of Rheumatology, Department of Internal Medicine, St. Vincent's Hospital, College of Medicine, The Catholic University of Korea, Seoul, Republic of Korea

* Both authors contributed equally to this work.

Supplementary Figures

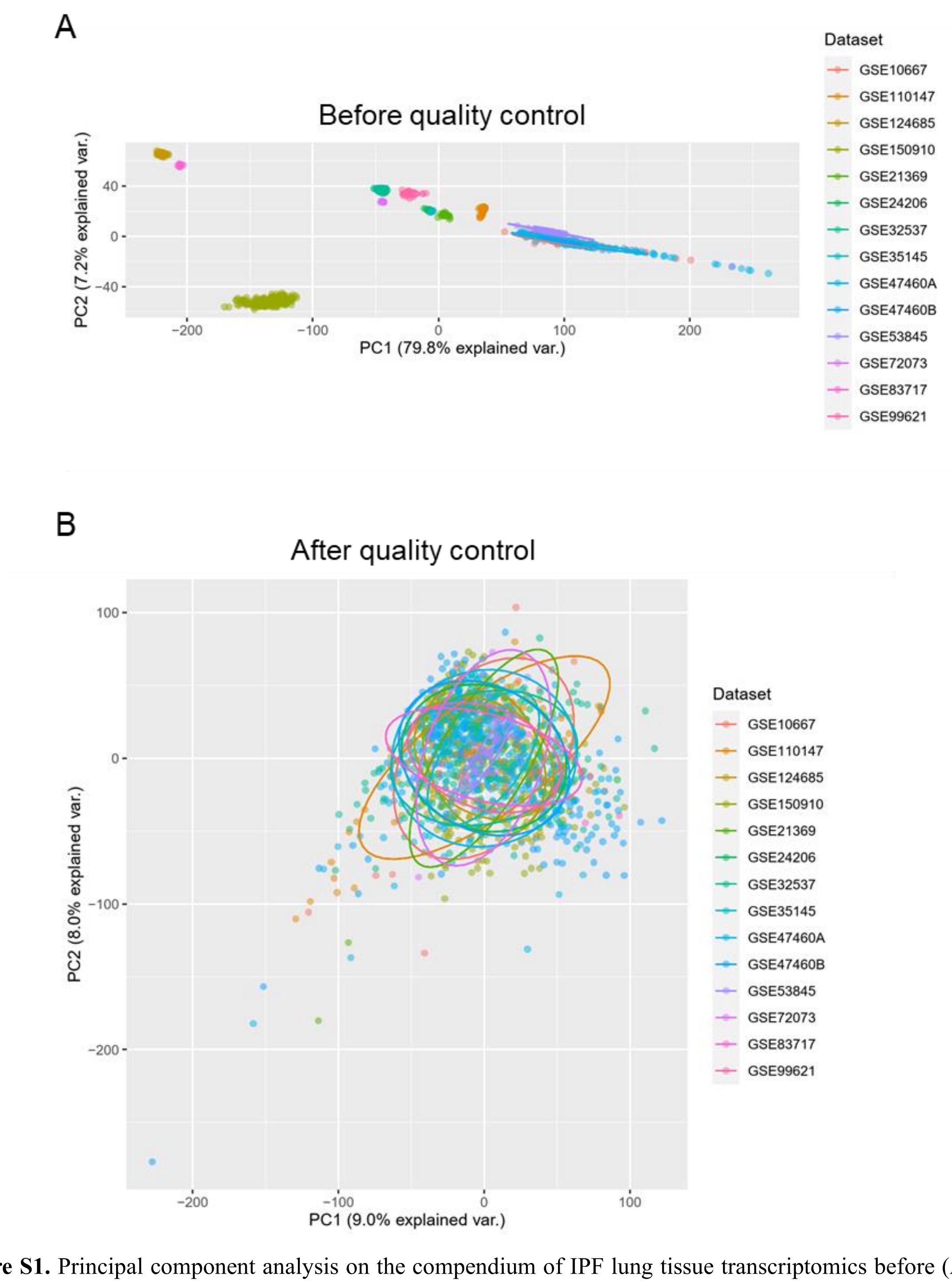

Figure S1. Principal component analysis on the compendium of IPF lung tissue transcriptomics before (A) and after (B) normalization and batch correction.

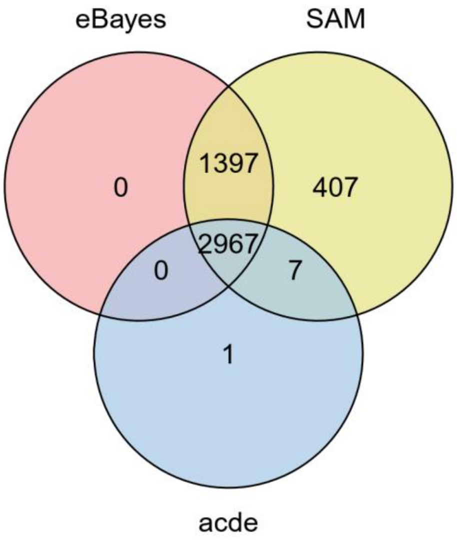

Figure S2. Refinement of the DEGs. Three independent methods were employed: (a) an empirical Bayesian method (eBayes) using the Benjamini-Hochberg procedure with adjusted p-value <0.01 as the significance threshold (R package limma); (b) the Significance Analysis of Microarray (SAM) method, with false discovery rate (FDR) <0.01 as the significance threshold (R package EMA); (c) multivariate inferential analysis method, with false discovery rate (FDR) <0.01 as the significance threshold (R package acde). An absolute value of fold change > 1.5 was considered as DEGs. The resulting list of upregulated DEGs (n=2,967) is the intersection of the three individual DEGs sets for each method to minimize the FDR statistic.

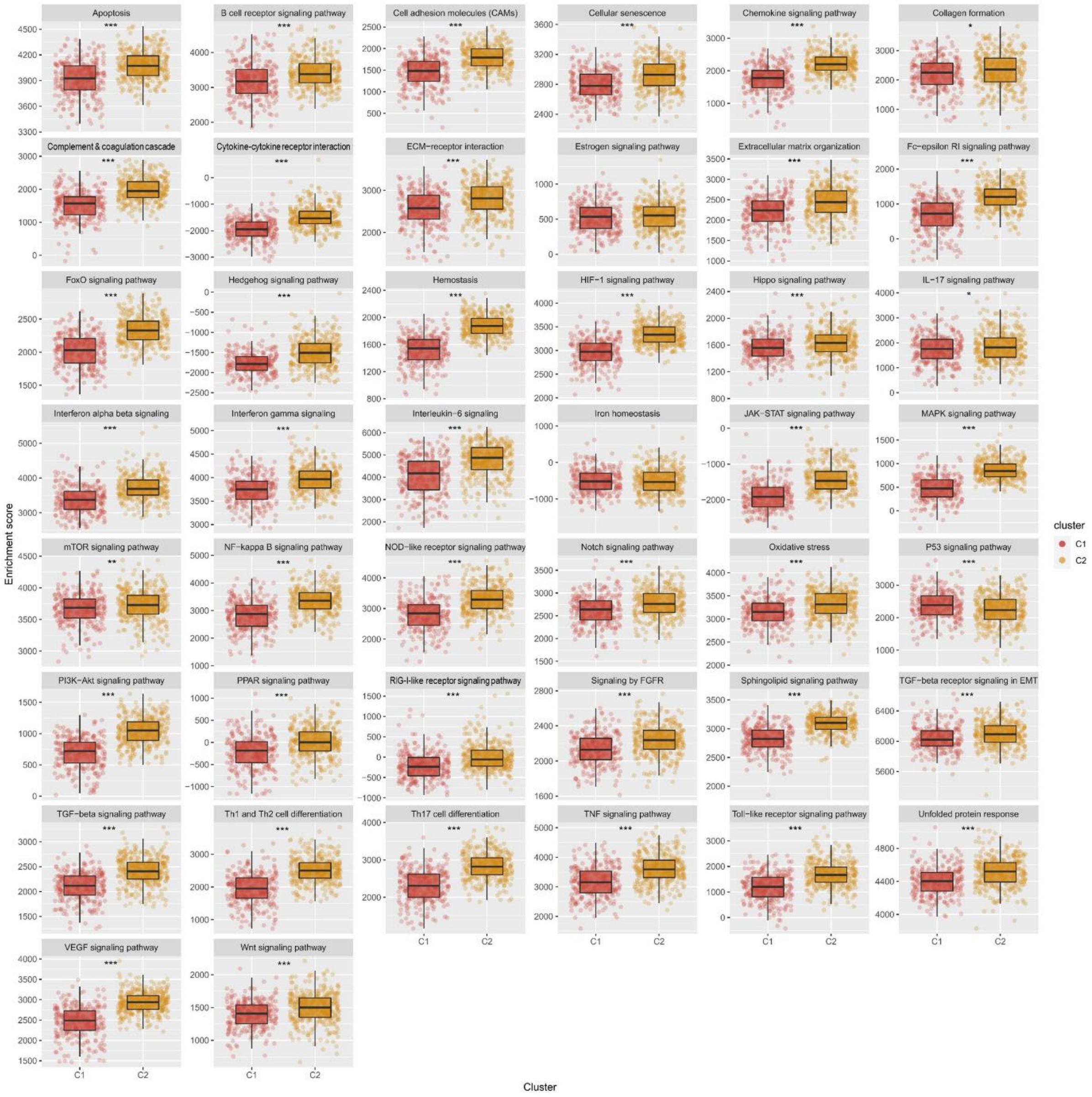

Figure S3. Pathway enrichment scores according to IPF subgroups. Gene-set information on signaling pathways or biological processes was obtained from KEGG and the Reactome database and single sample version of gene-set enrichment analysis (ssGSEA) was used to calculate an enrichment score. Differences across the two subgroups were evaluated using an unpaired *t*-test. *: $P < 0.01$; **: $P < 0.01$; ***: $P < 0.001$.

ECM, extracellular matrix; EMT, epithelial-mesenchymal transition; FGFR, fibroblast growth factor receptors; HIF, hypoxia-inducible factor; IL, interleukin; MAPK, Mitogen-activated protein kinase; RIG, retinoic acid-inducible gene; PPAR, peroxisome proliferator-activated receptors; TGF, transforming growth factor; Th, helper T cell; TNF, tumor necrosis factor; VEGF, vascular endothelial growth factor.

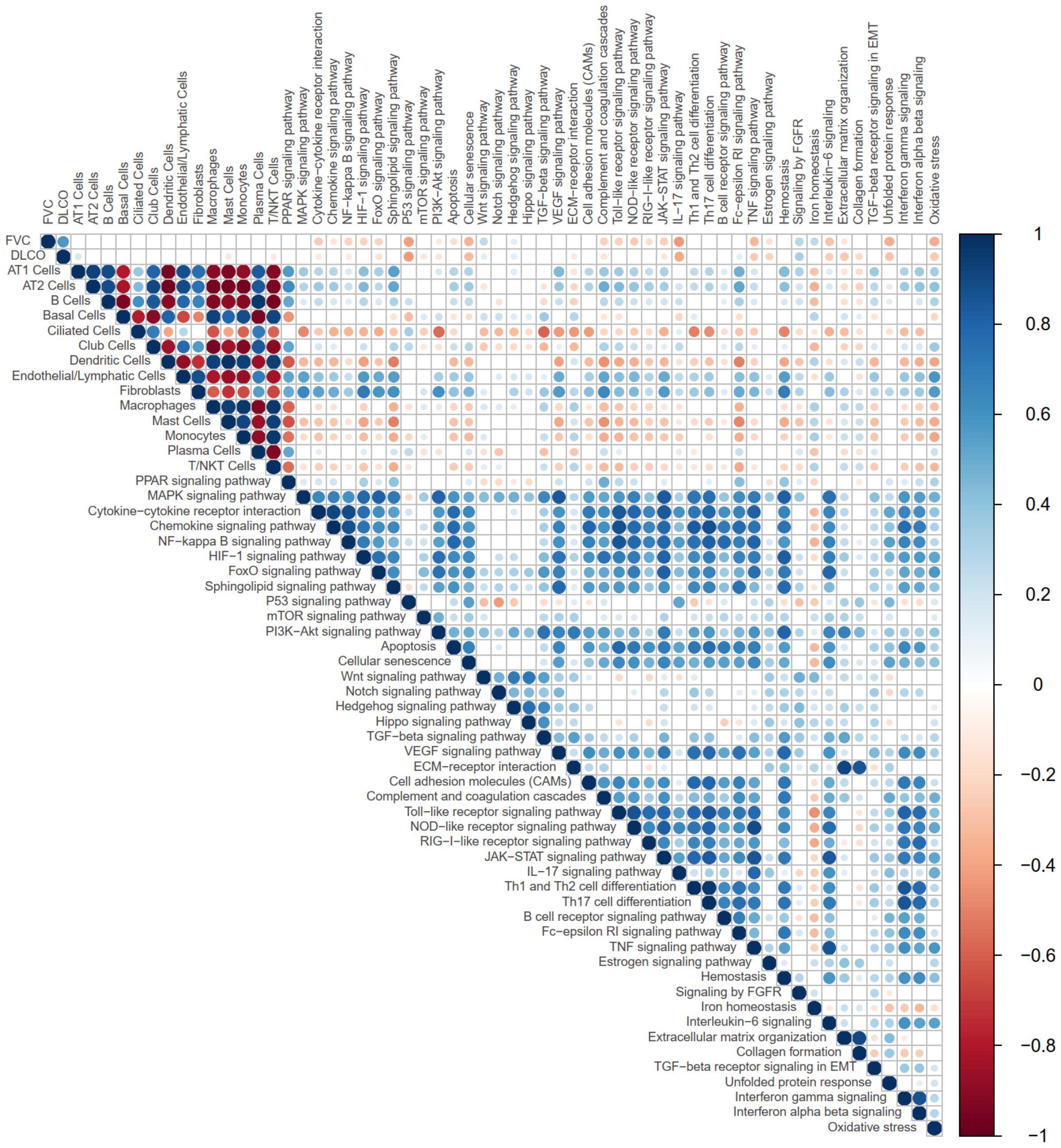

Figure S4. Correlation between pulmonary function parameters (FVC, DLCO), pathway and cell subset enrichment score. Correlation analysis was done by Pearson's method. Strong positive correlation was indicated by the blue hues, and negative by red hues. Significant correlation was filled by colors and insignificant correlation was blank.

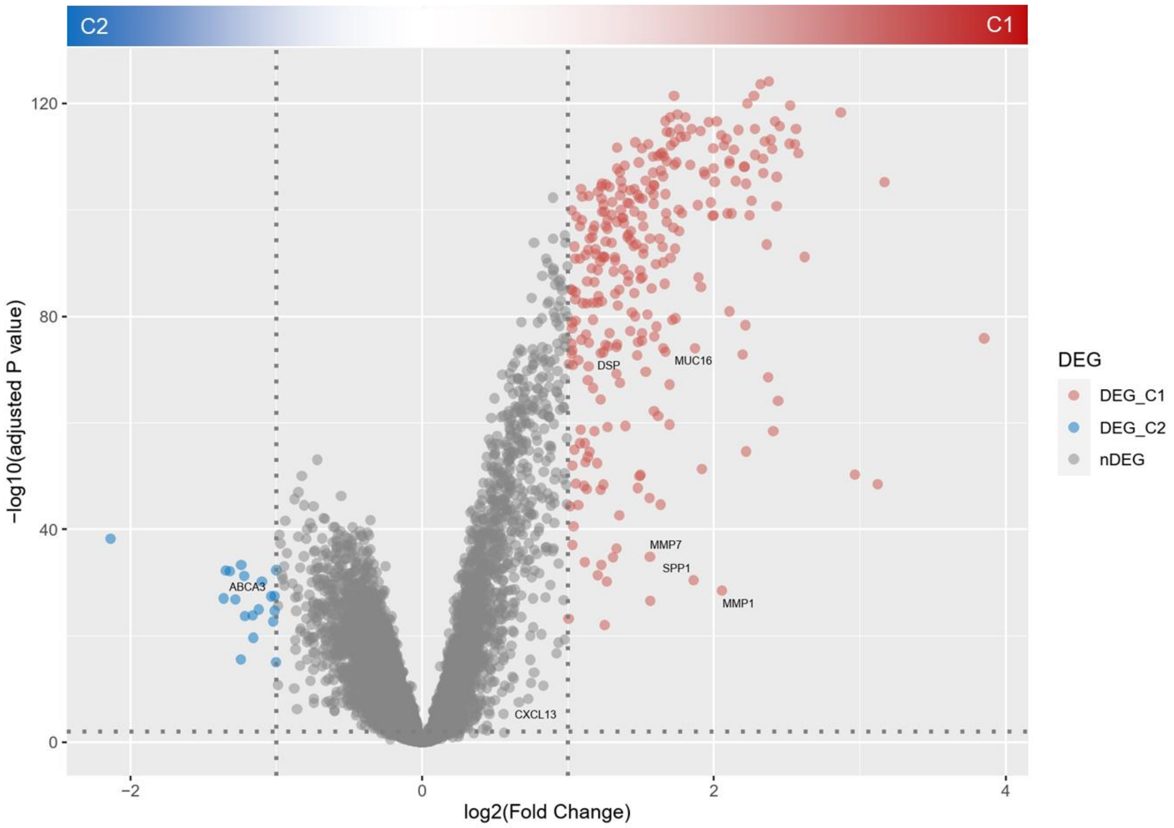

Figure S5. Volcano plot of expressed genes between two subgroups. Differentially expressed genes (DEGs) were filtered using R limma package and were defined as fold change > 2 and adjusted P value < 0.01. Upregulated DEGs of C1 and C2 were colored by red and blue hues, respectively.

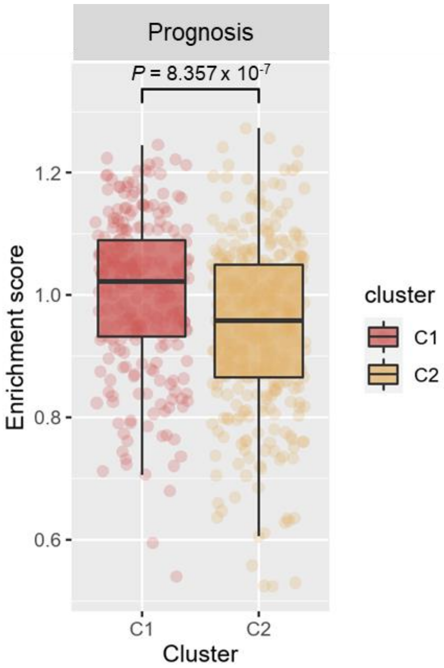

Figure S6. Enrichment scores of prognostic markers according to IPF subgroups by gene-set enrichment analysis. The difference between two subgroups was evaluated using unpaired *t*-test.
